# Infant Nutritional Status, Feeding Practices, Enteropathogen Exposure, Socioeconomic Status, and Illness Are Associated with Gut Barrier Function As Assessed by the Lactulose Mannitol Test in the MAL-ED Birth Cohort

**DOI:** 10.4269/ajtmh.16-0830

**Published:** 2017-05-30

**Authors:** Gwenyth O. Lee, Benjamin J. J. McCormick, Jessica C. Seidman, Margaret N. Kosek, Rashidul Haque, Maribel Paredes Olortegui, Aldo A. M. Lima, Zulfiqar A. Bhutta, Gagandeep Kang, Amidou Samie, Caroline Amour, Carl J. Mason, Tahmeed Ahmed, Pablo Peñataro Yori, Domingos B. Oliveira, Didar Alam, Sudhir Babji, Pascal Bessong, Estomih Mduma, Sanjaya K. Shrestha, Ramya Ambikapathi, Dennis R. Lang, Michael Gottlieb, Richard L. Guerrant, Laura E. Caulfield, for the MAL-ED Network Investigators

**Affiliations:** 1 Fogarty International Center, National Institutes of Health, Bethesda, Maryland; 2 Department of Epidemiology, University of Michigan School of Public Health, Ann Arbor, Michigan; 3 Department of International Health, Johns Hopkins Bloomberg School of Public Health, Baltimore, Maryland; 4 icddr,b, Dhaka, Bangladesh; 5 Asociacion Benefica PRISMA, Investigaciones Biomedicas, Iquitos, Peru; 6 Institute of Biomedicine, Federal University of Ceara, Fortaleza, Brazil; 7 Center of Excellence in Women and Child Health, the Aga Khan University, Karachi, Pakistan; 8 Division of Gastrointestinal Sciences, Christian Medical College, Vellore, India; 9 Department of Microbiology, University of Venda, Thohoyandou, South Africa; 10 Haydom Lutheran Hospital, Haydom, Tanzania; 11 Walter Reed/Armed Forces Research Institute of Medical Sciences, Kathmandu, Nepal; 12 Foundation for the NIH, Bethesda, Maryland; 13 Division of Infectious Diseases, University of Virginia, Charlottesville, Virginia

## Abstract

The lactulose mannitol (LM) dual sugar permeability test is the most commonly used test of environmental enteropathy in developing countries. However, there is a large but conflicting literature on its association with enteric infection and host nutritional status. We conducted a longitudinal cohort using a single field protocol and comparable laboratory procedures to examine intestinal permeability in multiple, geographically diverse pediatric populations. Using a previously published systematic review to guide the selection of factors potentially associated with LM test results, we examined the relationships between these factors and mucosal breach, represented by percent lactulose excretion; absorptive area, represented by percent mannitol excretion; and gut barrier function, represented by the L/M ratio. A total of 6,602 LM tests were conducted in 1,980 children at 3, 6, 9, and 15 months old; percent lactulose excretion, percent mannitol excretion, and the L/M ratio were expressed as age- and sex-specific normalized values using the Brazil cohort as the reference population. Among the factors considered, recent severe diarrhea, lower socioeconomic status, and recent asymptomatic enteropathogen infections were associated with decreased percent mannitol excretion and higher L/M ratios. Poorer concurrent weight-for-age, infection, and recent breastfeeding were associated with increased percent lactulose excretion and increased L/M ratios. Our results support previously reported associations between the L/M ratio and factors related to child nutritional status and enteropathogen exposure. These results were remarkably consistent across sites and support the hypothesis that the frequency of these exposures in communities living in poverty leads to alterations in gut barrier function.

## INTRODUCTION

The lactulose mannitol (LM) test is the most commonly used test of intestinal permeability and environmental enteropathy (EE) among children in the developing world.^1^ This noninvasive test measures the absorption and urinary excretion of two sugars following oral administration: lactulose, a large disaccharide, and mannitol, a smaller monosaccharide. Lactulose excretion reflects abnormal intestinal permeability to large molecules due to compromised tight junctions or areas of mucosal epithelial breach.^2^ Mannitol is thought to be absorbed across the cell membrane via a nonspecific epithelium transporter, and lower excretion indicates decreased intact epithelial surface area.^2^ Thus, a higher ratio of excreted lactulose to mannitol can result from increased lactulose excretion, decreased mannitol excretion, or both and is interpreted as an indicator of poorer intestinal barrier function. The L/M ratio is considered more robust than either probe individually; both lactulose and mannitol excretion may be influenced by factors such as intestinal transit time, gastric dilution, or bacterial degradation.^3^ However, several reports have noted that associations between the LM test and factors such as child anthropometric status are often driven by one probe, and thus it has also been suggested that these sugars should be considered both individually and in combination.^4^^,^^5^

The hypothesis that intestinal barrier function is altered among individuals frequently exposed to enteropathogens is supported by reports that L/M ratios (or related lactulose/rhamnose ratios) are elevated among both indigenous and visiting, apparently healthy adults in tropical countries compared with those from temperate climates.^6^^,^^7^ A number of studies have reported associations between the L/M ratio and factors such as recent diarrhea,^8^^–^^13^ or specific enteropathogen exposures including *Giardia*, *Cryptosporidium*, and rotavirus.^11^^–^^18^ Several studies have also reported associations between L/M ratios and child attained growth and nutritional status, as captured by concurrently measured child anthropometry (generally height for age *Z* score [HAZ] or weight for age *Z* score [WAZ]).^9^^,^^11^^–^^13^^,^^19^^–^^21^ However, associations between specific factors and L/M ratios are often inconsistent, and there are recognized difficulties in comparing L/M ratios across populations, given an absence of standardized protocols for urine collection (e.g., fasting time, duration of urine collection, and dosing) and standardized laboratory methods for probe detection using the currently most common high-performance liquid chromatography (HPLC) analytic platform.^22^

The Etiology, Risk Factors, and Interactions of Enteric Infections and Malnutrition and the Consequences for Child Health and Development Project (MAL-ED) is a multisite birth cohort study.^23^ The primary hypothesis of the study was that enteric infections and diet mediate growth in part through intestinal function, as measured by both the L/M ratio and fecal biomarkers of intestinal inflammation.^24^^,^^25^ Here, we describe the associations of morbidity, enteropathogen infections, infant feeding, and child nutritional status, as represented by anthropometry (WAZ), with the LM test, across sites and over time.

## MATERIALS AND METHODS

As previously described,^23^ the MAL-ED study is a prospective longitudinal birth cohort study conducted at eight sites: Dhaka, Bangladesh (BGD); Vellore, India (INV); Bhaktapur, Nepal (NEB); Naushero Feroze, Pakistan (PKN); Fortaleza, Brazil (BRF); Loreto, Peru (PEL); Venda, South Africa (SAV); and Haydom, Tanzania (TZH). Each site aimed to follow 200 newborns through the first 24 months of life. Overall 2,145 newborns were enrolled with the following inclusion criteria: < 17 days old, singleton, birth weight > 1,500 g, no serious illness, mother ≥ 16 years old, and plans to stay in the community at least 6 months. Written consent was obtained at enrollment and study procedures received ethical approval at each site and at collaborating institutions. Study participants were enrolled on a rolling basis over a 2.25-year period starting in October 2009.

Enrollment questionnaires captured sociodemographic, maternal, and household characteristics. Infants were weighed and measured using standardized procedures at enrollment and every month thereafter.^26^ Caregivers were queried biweekly regarding morbidity and feeding patterns.^26^^,^^27^ Non-diarrheal stools were collected monthly and analyzed for a panel of bacterial, viral, and parasitic enteropathogens; diarrheal stools were similarly evaluated. At 6-month intervals, information on water, sanitation conditions in the household, family income, and assets was obtained.

At 3, 6, 9, and 15 months, children were administered the LM test using a standardized protocol previously described.^25^ In brief, an LM solution (250 mg/mL lactulose and 50 mg/mL mannitol, 1,002 mOsm/L) was administered at 2 mL/kg of child weight up to a maximum of 20 mL. Just as is the case for oral medication, such as vitamins or probiotics for colic, this is not considered a breach in exclusive breastfeeding practice by health professionals. Children were fasted for 2 hours prior to and 30 minutes following solution administration, except breast milk, which was permitted ad libitum. The child's urine was collected continuously for 5 hours after sugar solution administration and the total urine volume was recorded.

Five sites (BRF, NEB, PKN, TZH, and SAV) used the HPLC system at the BRF laboratory, PEL used a commercially available liquid chromatography/mass spectrometry (LC-MSMS) system (Oregon Analytics Eugene, OR), and two sites (BGD and INV) used their own HPLC systems to measure urinary concentrations of lactulose and mannitol. Standardized protocols and quality control activities have been previously described.^22^^,^^28^ Results with the following issues were excluded: test administered outside ±30 days of the scheduled date, no mannitol or lactulose peak detected, calculated mannitol or lactulose excretion > 100%, urine collection time significantly longer (> 5.5 hours) or shorter (< 4.5 hours) than protocol, or other specific problems (e.g., the child vomited). From 7,461 urine collections, 859 (11.5%) were removed, most commonly for shortened collection times or insufficient quantities.^28^

All LM variables (L/M ratio, percent lactulose excretion, and percent mannitol excretion) were transformed (L/M ratio, normalized by age and sex and treating the Brazil (BRF) cohort as the reference population [LMZ], percent lactulose excretion, normalized by age and sex and treating the Brazil (BRF) cohort as the reference population [%Lac-Z], and percent mannitol excretion, normalized by age and sex and treating the Brazil (BRF) cohort as the reference population [%Man-Z]) so that alterations in intestinal barrier function were expressed as *Z* scores relative to the Brazil population. BRF was chosen as the reference for this purpose because children in this cohort showed the least growth faltering of the sites and were therefore considered the least likely to have adverse gut function. Briefly, percent lactulose, percent mannitol, and L/M ratio values at the Brazil site were stratified by age and sex, and then Box–Cox transformed to approximate normality. The same parameters from the Box–Cox transformation taken for the BRF site were then applied to variables from each other site, and the resulting values were scaled relative to the distribution of the BRF data (i.e., (*X* − μ_BRF, age, sex_)/δ_BRF, age, sex_).^28^ The full equations required to convert between raw LM test results and *Z* scores and reference tables have been published by Kosek and others.^28^

A systematic literature review of the LM test published from 2000 to 2010^1^ guided selection of factors for evaluation, including child nutritional status, socioeconomic status (SES), recent reported illness [diarrheal episodes and non-enteric fever (maternally reported fever absent diarrhea symptoms)], antibiotic use, breastfeeding and consumption of solids, symptomatic and asymptomatic enteropathogen detections, and season. Factors that were not analyzed include human immunodeficiency virus status, malaria parasitemia (infrequent in most sites), and plasma micronutrient status, which will be the subject of a future report. In most cases, the studies reviewed provided evidence of association between the factor of interest and LM, but causality could not be inferred.

Weight and length measures were converted to *Z* scores^29^; WAZ, length for age *Z* score (LAZ), and weight for length *Z* score (WLZ) measured concurrently with the LM test were considered. Anthropometry collected within ±14 days of the LM test was included (length data from PKN were excluded due to quality control issues). Enrollment WAZ was also evaluated.

SES variables were summarized into a Water Assets Maternal education and Income (WAMI) index,^30^ created to facilitate cross site comparisons within the MAL-ED study, which ranges in value from 0 to 1, where 0 represents the lowest possible SES and 1 the highest. Variables related to water and sanitation such as improved flooring, poultry ownership, and a hygiene score based on three questions related to handwashing practices^31^ were also evaluated.

For each LM test, the time since the last episode of diarrhea, non-enteric fever, and antibiotic course was calculated.^27^

Infant feeding variables were summarized to depict the percent of days with any breastfeeding or solid food consumption reported in the 7 days preceding the LM test, as well as the proportion of days during the prior 3-month period with exclusive or full breastfeeding (breast milk + water or water-based juices).^32^^,^^33^ All associations between feeding variables were also stratified by age (Supplemental Table 2).

Summary variables of enteropathogen exposure were included as the total pathogens identified in the time-matched stool.^34^^,^^35^ Associations between the LM test and the total number of viruses, bacteria, and parasites detected in all months prior to the test (e.g., total accumulated number of detected pathogens) were also evaluated.

Random-effects tobit regression was used to model associations between individual factors of interest and %Lac-Z, %Man-Z, and LMZ. Tobit models yield unbiased estimates when the dependent variable is truncated; these *Z* scores were right censored above ≥ 1.28. Random intercepts were included to account for repeated measures from the same child. All models adjusted for age, sex, site (BRF as the reference), and urine volume (centered and normalized as [ln(urine volume)] − mean[ln(urine volume)]/standard deviation[ln(urine volume)]). Urine volume explained 6.3% and 3.6% of the variance in %Lac-Z and %Man-Z, respectively (based on the pseudo-*R*^2^ of tobit regression models without additional parameters and pooling sites), and was also significantly associated with LMZ, although it explained less than 0.1% of the variance.

Each factor (e.g., WAZ and seasonality) was first considered in a simplified multivariable model adjusted for age, sex, site, and urine volume; significant factors were included in final multivariable models. Interaction terms between age and each major factor were individually tested, and those that decreased the model Akaike information criterion by ≥ 4 were considered “significant.”^36^ Evidence of seasonality was tested by running separate site-specific models and considering a partial Fourier series with harmonics of annual and biannual frequency (i.e., sine and cosine terms were included in the overall models included along with interaction terms between each of these variables and site^37^).

Sensitivity analyses were conducted by stratifying by age, sex, or site, and using outcomes of log transformed L/M ratio, percent lactulose, and percent mannitol. Because results using these outcomes and the LM *Z* scores were similar, only the *Z* score models are reported here.

In separate analyses, the time since the last diarrheal episode, non-enteric fever, or antibiotic course and LMZ, %Lac-Z, and %Man-Z was modeled using fractional polynomials, which is a simple but flexible approach to examining potentially nonlinear relationships between variables.^38^ In this case, we were interested in exploring whether there was any evidence of transient elevation in L/M ratios following an episode of illness. Because preliminary analysis suggested no relationship between lifetime-reported illness and the L/M ratio, only LM tests with ≥ 1 episode of diarrhea in the 90 days prior were considered, and each random-effects tobit regression model was adjusted for WAZ, age, sex, site, and urine volume. Models with diarrhea were adjusted for the severity of the episode using a previously described modified Vesikari score.^35^

## RESULTS

Selected characteristics of the children by age at LM test are reported in Table 1

**Table 1:** Distribution of factors by age

	All	3 months	6 months	9 months	15 months
Overall *N*	6,602	1,715	1,673	1,632	1,582
Geometric mean L:M (95% CI)	0.111 (0.109, 0.114)	0.123 (0.118, 0.129)	0.113 (0.108, 0.118)	0.109 (0.104, 0.114)	0.100 (0.095, 0.106)
Mean LMZ (SD)	0.37 (1.01)	0.10 (0.95)	0.38 (0.89)	0.61 (1.02)	0.41 (1.11)
Geometric mean % lactulose excretion	0.220 (0.214, 0.225)	0.276 (0.263, 0.289)	0.226 (0.216, 0.237)	0.201 (0.190, 0.212)	0.183 (0.173, 0.194)
Mean Lac-Z	0.99 (1.41)	0.78 (1.43)	1.09 (1.47)	0.98 (1.21)	1.23 (1.50)
Geometric mean % mannitol excretion	1.892 (1.835, 1.951)	2.132 (2.008, 2.262)	1.913 (1.808, 2.025)	1.774 (1.669, 1.887)	1.756 (1.641, 1.878)
Mean Man-Z	0.38 (1.11)	0.38 (1.02)	0.36 (1.06)	0.29 (1.05)	0.52 (1.29)
Mean WAZ at time of LM measurement	−0.66 (1.27)	−0.59 (1.26)	−0.54 (1.25)	−0.66 (1.27)	−0.87 (1.25)
Any breastmilk (%)	92.0	97.8	95.3	92.7	81.5
Any solids (%)	74.8	26.9	77.8	98.2	99.6
WAMI	0.56 (0.22)	–	–	–	–
Recent diarrhea (%)	13.6	11.6	15.1	14.6	13.0
Median time from last diarrheal episode to LM (10th, 90th percentile)	48 (5, 173)	23 (2, 67)	34 (4, 107)	52 (5.5, 160)	76.5 (8, 270)
Median severity of last episode (10th, 90th percentile)	3 (1, 6)	3 (1, 6)	3 (1, 6)	3 (1, 6)	3 (1, 6)
Recent non-enteric fever (%)	12.0	11.5	11.7	13.0	12.0
Median time since last non-enteric fever episode	30 (4, 113)	22 (3, 58)	27 (4, 98)	31 (4, 109)	41 (5, 185)
Recent antibiotic use (%)	18.9	14.5	21.7	21.6	18.1
Median time since last antibiotic use	33 (4, 136)	23 (4, 67)	27 (4, 101)	33 (4, 128)	50 (6, 224)
Median urine volume (10th, 90th percentile)	40 (13, 103)	50 (15, 108)	37 (12, 95)	35 (12, 92)	40 (15, 106)

CI = confidence interval; SD = standard deviation; LMZ = L/M ratio, normalized by age and sex and treating the Brazil (BRF) cohort as the reference population; LM = lactulose mannitol; WAZ = weight for age *Z* score; WAMI = Water Assets Maternal education and Income.

. The percent variability of the test results explained by within-child variability was 12.1%, 30.0%, and 21.9% for LMZ, %Lac-Z, and %Man-Z, respectively. Site explained 4.7%, 22.8%, and 14.4% of the variability in each result. There was little evidence that the association between specific factors and LMZ was modified by laboratory, site, age, or sex (Supplemental Table 1). Final models included terms for age, site, sex, urine volume, concurrent WAZ, seasonality, recent breastfeeding, recent solid food consumption, WAMI, and concurrent overall asymptomatic enteropathogen exposure (Table 2

**Table 2:** Summary factors in three models

	Mean (SD)/%/median (10th, 90th)	%Lac-Z	%Man-Z	LMZ
Mean WAZ at time of LM measurement	−0.66 (1.27)	−0.08 (−0.11, −0.04)	−0.01 (−0.04, 0.01)	−0.046 (−0.07, −0.020)
		*P* < 0.001	*P* = 0.331	*P* = 0.001
Any breastmilk	92.0%	0.35 (0.22, 0.48)	0.08 (−0.02, 0.19)	0.111 (0.008, 0.215)
		*P* < 0.001	*P* = 0.130	*P* = 0.035
Any solids	74.8%	0.04 (−0.08, 0.15)	0.17 (0.08, 0.26)	−0.071 (−0.158, 0.013)
		*P* = 0.532	*P* < 0.001	*P* = 0.115
WAMI	0.56 (0.22)	−0.01 (−0.27, 0.25)	0.41 (0.20, 0.62)	−0.397 (−0.599, −0.196)
		*P* = 0.948	*P* < 0.001	*P* < 0.001
Total pathogens in time-matched stool	1.05 (1.00)	0.01 (−0.02, −0.05)	−0.04 (−0.07, −0.01)	0.058 (0.029, 0.087)
		*P* = 0.436	*P* = 0.004	*P* < 0.001
Total lifetime pathogens/stool	0.75 (0.57)	−0.04 (−0.12, −0.03)	−0.05 (−0.11, 0.01)	0.027 (−0.031, 0.085)
		*P* = 0.268	*P* = 0.105	*P* = 0.816
Median urine volume (10th, 90th percentile)	40 (13, 103)	0.46 (0.42, 0.50)	0.28 (0.24, 0.31)	0.038 (0.006, 0.071)
		*P* < 0.001	*P* < 0.001	*P* = 0.020
Sex				
M	50.9%	Ref	Ref	Ref
F	49.1%	0.28 (0.21, 0.36)	0.21 (0.15, 0.27)	−0.079 (−0.135, −0.023)
		*P* < 0.001	*P* < 0.001	*P* = 0.006
Age				
3	26.0%	Ref	Ref	Ref
6	25.3%	0.39 (0.28, 0.50)	−0.04 (−0.13, 0.04)	0.376 (0.290, 0.462)
		*P* < 0.001	*P* = 0.332	*P* < 0.001
9	24.7%	0.47 (0.35, 0.59)	−0.06 (−0.16, 0.04)	0.562 (0.465, 0.660)
		*P* < 0.001	*P* = 0.226	*P* < 0.001
15	24.0%	0.51 (0.38, 0.64)	0.08 (−0.02, 0.18)	0.355 (0.254, 0.455)
		*P* < 0.001	*P* = 0.131	*P* < 0.001
Site				
BRF	11.5%	Ref	Ref	Ref
BGD	14.0%	0.08 (−0.12, 0.28)	0.43 (0.28, 0.59)	0.189 (0.042, 0.336)
		*P* = 0.450	*P* < 0.001	*P* = 0.012
INV	13.8%	0.14 (−0.07, 0.45)	0.90 (0.74, 1.07)	0.283 (0.129, 0.438)
		*P* = 0.186	*P* < 0.001	*P* < 0.001
NEB	13.8%	0.05 (−0.14, 0.23)	0.38 (0.24, 0.52)	−0.025 (−0.154, 0.104)
		*P* = 0.588	*P* < 0.001	*P* = 0.702
PEL	15.4%	0.02 (−0.16, 0.21)	0.13 (−0.02, 0.27)	0.560 (0.422, 0.698)
		*P* = 0.802	*P* = 0.087	*P* < 0.001
PKN	12.7%	−0.04 (−0.24, 0.15)	0.37 (0.21, 0.52)	0.401 (0.254, 0.549)
		*P* = 0.657	*P* < 0.001	*P* < 0.001
SAV	9.2%	−0.04 (−0.26, 0.18)	−0.49 (−0.64, −0.34)	0.565 (0.415, 0.715)
		*P* = 0.742	*P* = 0.516	*P* < 0.001
TZH	9.7%	−0.07 (−0.27, 0.12)	0.06 (−0.12, 0.24)	0.200 (0.024, 0.375)
		*P* = 0.462	*P* = 0.516	*P* = 0.036
Constant	na	−0.48 (−0.69, −0.27)	−0.15 (−0.32, 0.03)	−0.330 (−0.495, 0.164)
		*P* < 0.001	*P* = 0.098	*P* < 0.001

BRF = Fortaleza, Brazil; BGD = Dhaka, Bangladesh; INV = Vellore, India; %Lac-Z = percent lactulose excretion, normalized by age and sex and treating the Brazil (BRF) cohort as the reference population; LMZ = L/M ratio, normalized by age and sex and treating the Brazil (BRF) cohort as the reference population; %Man-Z = percent mannitol excretion, normalized by age and sex and treating the Brazil (BRF) cohort as the reference population; na = not applicable; NEB = Bhaktapur, Nepal; PEL = Loreto, Peru; PKN = Naushero Feroze, Pakistan; SAV = Venda, South Africa; SD = standard deviation; TZH = Haydom, Tanzania; WAMI = Water Assets Maternal education and Income; WAZ = weight for age *Z* score. The first column represents the distribution of the factor in the data; for instance, the mean WAZ at the time of the LM test was −0.66 and 92.0% of children had breastmilk in the week prior.

). After adjusting for these factors, the percent variability explained by child and site fell to 10.6% and 3.7% (LMZ); 20.9% and 13.9% (%Lac-Z); and 19.9% and 11.6% (%Man-Z).

Seasonal trends were not attenuated after adjusting for recent diarrhea or pathogen exposure, suggesting that they were driven by other factors. There was heterogeneity between sites in the timing of seasonal peaks as well as whether seasonal trends in %Lac-Z and %Man-Z coincided or not (Supplemental Figure 1).

Although LAZ, WAZ, and WLZ were all inversely associated with LMZ, only WAZ was retained for the full model. Sensitivity analyses (excluding the PKN site) suggested that the inclusion of WAZ led to better model fit than either LAZ and WLZ individually, or in combination. In these analysis, there was little evidence that LAZ and WLZ acted differently on LMZ, rather, WAZ, LAZ, and WLZ were inversely associated with LMZ but WAZ appeared to capture the relationship best. Associations between child nutritional status (anthropometry) and LMZ were driven by percent lactulose excretion (WAZ was inversely associated with %Lac-Z, rather than %Man-Z). There was no evidence that this relationship was modified by age or sex. There was no association between enrollment weight and LMZ.

Overall, any breastfeeding in the past week was associated with greater LMZ, and this was due to percent lactulose excretion (i.e., greater %Lac-Z). In contrast, consumption of solids in the past week was associated with lower LMZ, primarily due to higher percent mannitol excretion (i.e., greater %Man-Z). Although both the prevalence of breastfeeding and consumption of solids varied with age, there was no evidence of interactions between feeding patterns and age.

**Figure 1.**
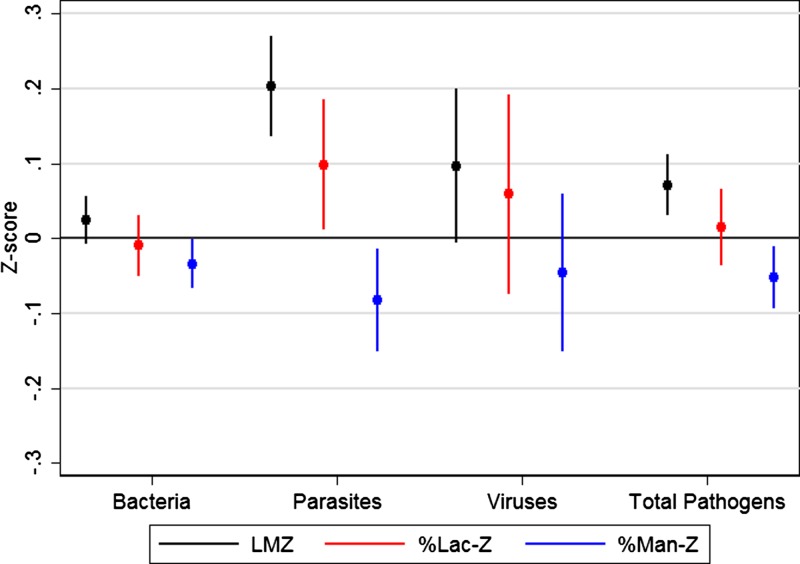
Predicted LMZ by enteropathogen exposure. This figure represents the results of models in which concurrent enteropathogens (detected in the matched asymptomatic stool) were related to %Lac-Z, %Man-Z, and LMZ. Total bacteria includes *Aeromonas*, *Campylobacter*, enteroaggregative Ecoli (EAEC), enteroinvasive Ecoli (EIEC), atypical and typical enteropathogenic Escherichia coli (Atypical EPEC and EPEC), heat-stable- and heat-lable -enterotoxigenic Ecoli (ST-ETEC and LT-ETEC), *Plesiomonas shigilloides*, *Salmonella, Shigella*, STEC, *Vibrio*, and *Yersinia enterocolitica*; total parasites includes: *Ascaris lumbricoides*, *Balantidium coli*, *Chilomastix mesnili*, *Cryptosporidium*, *Cyclospora*, *Entamoeba histolytica*, *Endolimax nana*, *Enterobius vermicularis*, *Giardia lamblia, Hymenolepis diminuta*, *Hymenolepis nana*, Hookworm, *Iodamoeba bütschlii*, *Isospora*, *Strongyloides stercoralis*, *Schistosoma*, *Trichuris trichiura*, and *Taenia solium*. Total viruses included astrovirus, rotavirus, and adenovirus (norovirus genotype I [GI] and GII were excluded because they were not tested in all asymptomatic stools). %Lac-Z = percent lactulose excretion, normalized by age and sex and treating the Brazil (BRF) cohort as the reference population; LMZ = L/M ratio, normalized by age and sex and treating the Brazil (BRF) cohort as the reference population; %Man-Z = percent mannitol excretion, normalized by age and sex and treating the Brazil (BRF) cohort as the reference population.

A greater number of enteropathogens in the time-matched asymptomatic stool were associated with higher LMZ, as was the total number of parasites detected (Figure 1

). Neither the total number of bacteria nor viruses detected in the time-matched asymptomatic stool were statistically significantly associated with LMZ; however, the total enteropathogen number yielded a better model fit than total parasites alone. There was no evidence that the association between enteropathogens and LMZ varied by age.

**Figure 2.**
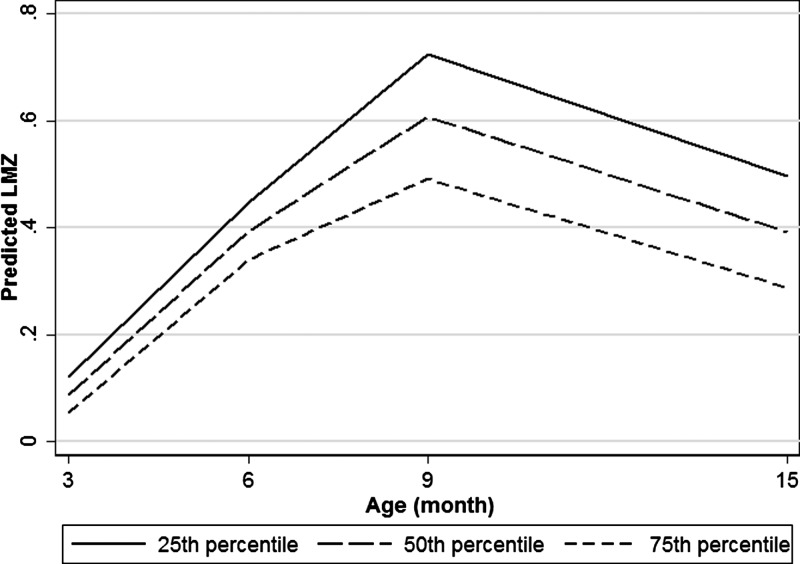
Predicted LMZ by WAMI. This figure represents the predictions of the LMZ model across levels of age and WAMI (socioeconomic) index. There was a statistically significant interaction between age and socioeconomic status. As children aged, the model predicted an increasing divergence in LMZ between children with higher vs. lower socioeconomic status. LMZ = L/M ratio, normalized by age and sex and treating the Brazil (BRF) cohort as the reference population; WAMI index = Water Assets Maternal education and Income index.

The WAMI score was inversely associated with LMZ, with a 1 unit increase in WAMI (from lowest to highest) associated with a 0.43 lower LMZ. There was a statistically significant interaction between WAMI and age, with the greatest effect of WAMI on LMZ occurring among older (9- and 15-month-old) children (Figure 2

). Individual WAMI components were also associated with lower LMZ (Table 3

**Table 3:** Regression models by socioeconomic status and WASH variables

	%Lac-Z	%Man-Z	LMZ
WAMI component 1: unimproved water and sanitation	Ref	Ref	Ref
WAMI component 1: improved water or sanitation	0.148 (−0.319, 0.023)	−0.099 (−0.247, 0.049)	0.058 (−0.088, 0.20)
	*P* = 0.031	*P* = 0.189	*P* = 0.434
WAMI component 1: improved water and sanitation	−0.222 (−0.412, −0.031)	−0.19 (−0.183, 0.144)	−0.063 (−0.22, 0.10)
	*P* = 0.023	*P* = 0.817	*P* = 0.440
WAMI component 2: asset score (0–8)	−0.096 (−0.125, −0.066)	0.028 (0.011, 0.044)	−0.019 (−0.034, −0.003)
	*P* = 0.463	*P* = 0.001	*P* = 0.017
WAMI component 3: income category (0–8)*	0.002 (−0.016, 0.021)	0.020 (0.005, 0.035)	−0.020 (−0.034, −0.005)
	*P* = 0.798	*P* = 0.010	*P* = 0.007
WAMI component 4: maternal education (0–8)†	0.013 (−0.007, 0.033)	0.027 (0.010, 0.044)	−0.021 (−0.037, −0.005)
	*P* = 0.187	*P* = 0.002	*P* = 0.011
Overall WAMI (0–1)	0.026 (−0.212, 0.265)	0.410 (0.209, 0.611)	−0.376 (−0.570, −0.182)
	*P* = 0.828	*P* < 0.001	*P* < 0.001
Improved sanitation (ref = unimproved)	−0.094 (−0.123, −0.064)	−0.008 (−0.033, 0.017)	−0.067 (−0.092, −0.043)
	*P* < 0.001	*P* = 0.529	*P* < 0.001
Improved drinking water (ref = unimproved)	−0.066 (−0.217, 0.084)	0.024 (−0.107, 0.154)	−0.036 (−0.165, 0.092)
	*P* = 0.390	*P* = 0.720	*P* = 0.580
Floor = dirt	Ref	Ref	Ref
Cement, wood, vinyl, or other	−0.046 (−0.122, 0.029)	0.060 (−0.017, 0.138)	0.024 (−0.068, 0.116)
	*P* = 0.231	*P* = 0.128	*P* = 0.613
Chicken ownership (Y/N)	−0.010 (−0.098, 0.078)	0.008 (−0.063, 0.080)	−0.024 (−0.092, 0.0440
	*P* = 0.822	*P* = 0.824	*P* = 0.492
Siblings			
None	Ref	Ref	Ref
2–4	0.056 (−0.022, 0.134)	−0.024 (−0.087, 0.040)	0.084 (0.024, 0.143)
	*P* = 0.161	*P* = 0.464	*P* = 0.006
≥ 5	0.090 (−0.030, 0.210)	−0.003 (−0.101, 0.0950	0.068 (−0.026, 0.162)
	*P* = 0.141	*P* = 0.952	*P* = 0.155
Hygiene score (range 0–12, high = less reported handwashing)	−0.011 (−0.028, 0.006)	−0.016 (−0.030, −0.002)	0.003 (−0.010, 0.016)
	*P* = 0.196	*P* = 0.022	*P* = 0.660

%Lac-Z = percent lactulose excretion, normalized by age and sex and treating the Brazil (BRF) cohort as the reference population; LMZ = L/M ratio, normalized by age and sex and treating the Brazil (BRF) cohort as the reference population; %Man-Z = percent mannitol excretion, normalized by age and sex and treating the Brazil (BRF) cohort as the reference population; WAMI = Water Assets Maternal education and Income; WASH = water, sanitation and hygiene risk; WAZ = weight for age *Z* score. Separate models for each WASH factor are given. Each model adjusts for WAZ, urine volume, site, age, and child sex.

* Reported income in U.S. dollars, categorized in all-site noniles.

† Years of maternal education divided by two.

). These associations were driven by greater percent mannitol excretion. Access to improved sanitation was statistically significantly associated with lower LMZ, but access to improved drinking water and flooring type was not.

**Figure 3.**
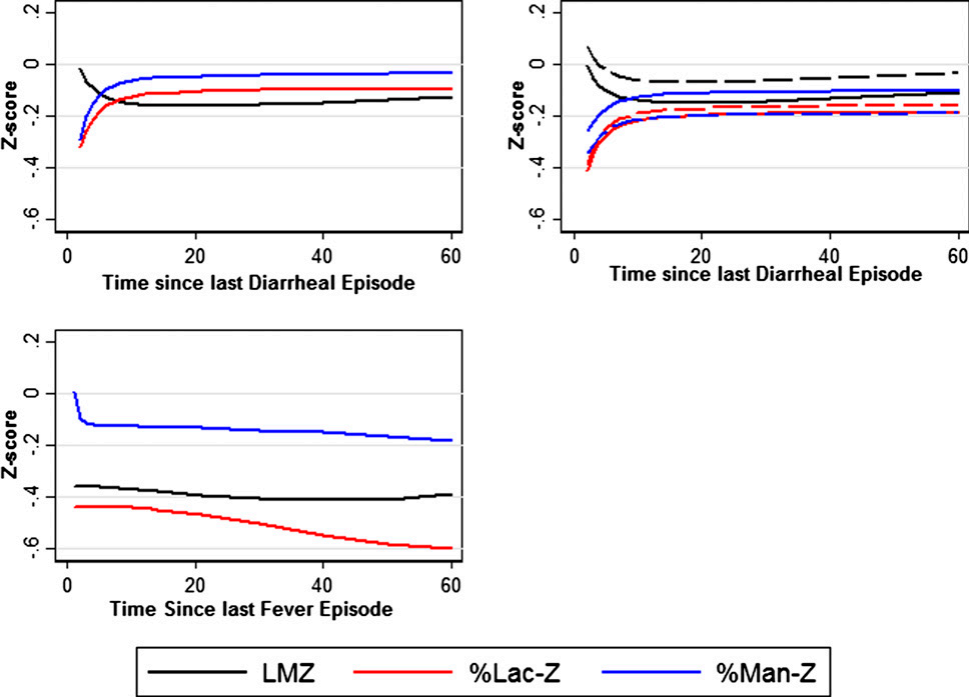
LMZ in relation to recent diarrheal illness and non-enteric fever. These figures show the predicted values of models in which fractional polynomial term were used to model the relationship between the LM test and the time since last diarrhea (T). Panel 1 (upper left) The predicted association between time since recent illness and LMZ where fractional polynomials were used to examine whether there was evidence of a nonlinear drop-off in LMZ following symptomatic illness. In Panel 1 (upper left) and Panel 2 (upper right) the fractional polynomial terms included in the model were log(T) and log(T)2; in the % Lac-Z and % Man-Z models, the fractional polynomial terms were T^−2^ and log(T)*T^−2^. In the fever LMZ model Panel 3 (lower left), the fractional polynomial terms for time since last diarrhea (T) were T^2^ and log(T)^2^, in the %Lac-Z these were T^3^ and log(T)^3^, and %Man-Z models, the fractional polynomial terms were T^−2^ and T^2^. LMZ = L/M ratio, normalized by age and sex and treating the Brazil (BRF) cohort as the reference population; %Lac-Z = percent lactulose excretion, normalized by age and sex and treating the Brazil (BRF) cohort as the reference population; %Man-Z = percent mannitol excretion, normalized by age and sex and treating the Brazil (BRF) cohort as the reference population.

LMZ was elevated immediately following an episode of diarrhea, and dropped rapidly in the 0–14 days following (Figure 3A

). This association was mostly due to a decrease in percent mannitol excretion. We also observed an association between recent severe diarrhea and elevated LMZ (Figure 3B). %Man-Z dropped rapidly following reported non-enteric fever, whereas %Lac-Z decreased gradually (Figure 3C). There was no association between the time since last reported antibiotic usage and LMZ, %Lac-Z, or %Man-Z.

## DISCUSSION

Using the eight diverse MAL-ED cohorts, we examined household, environmental, and individual factors that provide context for the interpretation of LM test results among pediatric populations in low- and middle-income countries. Here, we strictly consider associations with factors preceding or concurrent to the LM test.

We found that a higher L/M ratio was positively associated with breastfeeding, recent diarrheal illness, and enteropathogen burden, and negatively associated with higher SES and the consumption of solid food.

We observed inverse associations between LMZ and concurrent nutritional status (most strongly with WAZ). This is consistent with prior reports by Goto and others (driven by percent mannitol excretion),^11^^,^^19^ Lin and others (driven by both mannitol and lactulose excretion),^20^ and Campbell and others (driven primarily by lactulose excretion)^39^ who found that HAZ and WAZ, but not WHZ (which reflects acute rather than chronic malnutrition), were associated with the LM test. Others^12^^,^^21^ report no association between anthropometric status and the LM test. However, associations between cross-sectional child anthropometry and LM do not necessarily imply a role for gut permeability in compromising future child growth. Future work will examine the evidence for an association between LM, changes in LM over time, and subsequent changes in LAZ, WAZ, or child growth.

Our results also support previously reported associations between household water, sanitation, and hygiene (WASH)-related factors and the LM test.^20^ We found SES factors that are only indirectly related to WASH such as greater maternal education, higher income, and greater asset ownership to be inversely associated with the L/M ratio, even after adjusting for WASH factors (driven by both mannitol and lactulose excretion). Further work is needed to determine whether this relationship would remain intact with more sensitive pathogen detection methods.

The presence of any breastfeeding in the past week was associated with higher L/M ratios. In this cohort, only six (of 2,053) infants were never breastfed,^32^ and the median duration of exclusive breastfeeding ranged from 12 to 105 days (PKN/BGD), with most sites (seven of the eight sites) having median duration < 90 days (the time of the first LM test).^39^ At every time point when LM was tested (3–15 months), most children received both breast milk and solids in the week prior to the test (Table 1). However, this overall trends mask underlying within-individual heterogeneity in feeding practices across the study period, as re-initiation of exclusive breastfeeding was common in several sites.^39^ There was no association between LM and the proportion of days during the prior 3-month period with exclusive or full breastfeeding. Given these factors, the results we report here suggest an association between LM and the recent inclusion of breast milk in the diet, but not between LM and the overall style of feeding, among a largely not exclusively breastfed group of infants. Relationships between feeding variables and LM were also stratified by age (Supplemental Table 2), and interaction terms between age and feeding practice were considered, but these were not statistically significant. The relationship between breast milk and the LM test contrasts with studies that have found breastfeeding to be associated with lower L/M ratios compared with formula feeding among both term and preterm infants during the neonatal period.^40^^–^^42^ However, our study population was older (3- to 15-month-old infants) and may not be comparable. Lactulose is not found in breast milk, although it is found in low doses in ultra-high-temperature heat-treated milk.^43^ We did not observe any consistent association between consumption of animal milks, formula (very few MAL-ED infants ever received formula) and the LM test. Future work is needed to understand whether the LM test is influenced by breastfeeding during urine collection or by differences in transit time between breastfed and weaned infants.^44^

As previously reported,^45^ there was evidence of an association between LMZ and asymptomatic enteropathogen infections, and particularly between parasitic infections, which in our data were primarily protozoal (*Cryptosporidium* and *Giardia*) rather than soil-transmitted helminths. These relationships were driven by both elevated lactulose and decreased mannitol and are also supported by prior studies.^19^^,^^46^^,^^47^ We were underpowered to evaluate associations between the LM test and enteropathogen-specific diarrhea occurring proximal to the test (Supplemental Table 2); however, the severity of recent diarrhea was associated with higher L/M ratios, driven by decreased mannitol excretion.

Several factors (WAZ and recent breastfeeding) affected L/M ratios through lactulose excretion, others (recent consumption of solids) through mannitol excretion, and others (parasitic infections) through associations with both sugars. It was also notable that in some cases (e.g., chronic enteropathogen exposures), the excretion of both probes was altered, leading to no overall association with the L/M ratio. A recent study by Kelly and others found that, counterintuitively, greater villus surface area was also associated with increased macromolecular translocation as measured by percent lactulose excretion^48^; this may partially explain this result.

Apart from child nutritional status and lifetime enteropathogen exposure, factors associated with the LM test tended to be recent or acute exposures, rather than longer-term trends or patterns in feeding or reported illness. This finding together with the finding that the within-child intraclass correlation coefficient was ≤ 30% for %Lac-Z, %-Man-Z, and LMZ, suggests that L/M ratios may fluctuate over relatively short time scales. However, because acute factors were highly present among the cohort (e.g., approximately 11.5% of days were associated with a diarrheal episode in the 2 weeks prior, and 65% of asymptomatic stools had at least one pathogen detected^35^), it is likely that a significant percentage of time would be spent with L/M ratios abnormally elevated due to these exposures.

There are several limitations to our study. Our study protocol required all infants to be fasted for a minimum of 2 hours prior to and 30 minutes following the administration of the disaccharide solution, breast milk was an exception, as it was permitted ad lib. Given we observed a positive association between breastfeeding and LM, the lack of detailed information about breastfeeding events immediately prior to and during to the test is a limitation. Despite extensive efforts to standardize laboratory techniques for the determination of lactulose and mannitol across course of the study, which included ongoing quality control, the determination of HPLC is known to be challenging.^22^ For this reason, all analyses were also subject to sensitivity analyses where only the five sites run by a single laboratory (BRF, NEB, PKN, SAV, and TZH) were included. Another potential limitation was to use a population (the BRF cohort) as an internal reference from which to calculate LM *Z* scores. The BRF cohort was selected for this purpose because it experienced very little growth faltering relative to the WHO growth standards. However, children in the BRF cohort nevertheless experienced frequent asymptomatic enteric infections and the results we demonstrate here suggest that pathogen exposure was associated with increased LM. Future work should explore compensatory mechanisms that influence gut barrier function repair and/or protect against growth impairment in this context.

The EE hypothesis states that poor access to potable water and improved sanitation results in increased enteropathogen exposure and diarrheal illness, leading in turn to small intestine alterations that result in poorer infant growth and development. Here, we address the first half of this hypothesis, by reporting on associations between the L/M ratio and factors related to illness, enteropathogens, and water and sanitation. Our results suggest these factors influence the L/M ratio via either or both probes, and generally operate over a period of days to weeks rather than months. Overall, the strength and consistency of these results across eight diverse geographic sites support the use of the LM test as an indicator of altered gut function among children at risk of EE.

## Supplementary Material

Supplemental Tables and figures.
