# Supplementary material for: Infant Nutritional Status, Feeding Practices, Enteropathogen Exposure, Socioeconomic Status, and Illness Are Associated with Gut Barrier Function As Assessed by the Lactulose Mannitol Test in the MAL-ED Birth Cohort
Source: Am J Trop Med Hyg. 2017 May 30;97(1):281–90. doi: 10.4269/ajtmh.16-0830 (PMC5508897; doi:10.4269/ajtmh.16-0830)
Supplement: Supplementary file 1 [file tpmd160830.SD1.pdf]

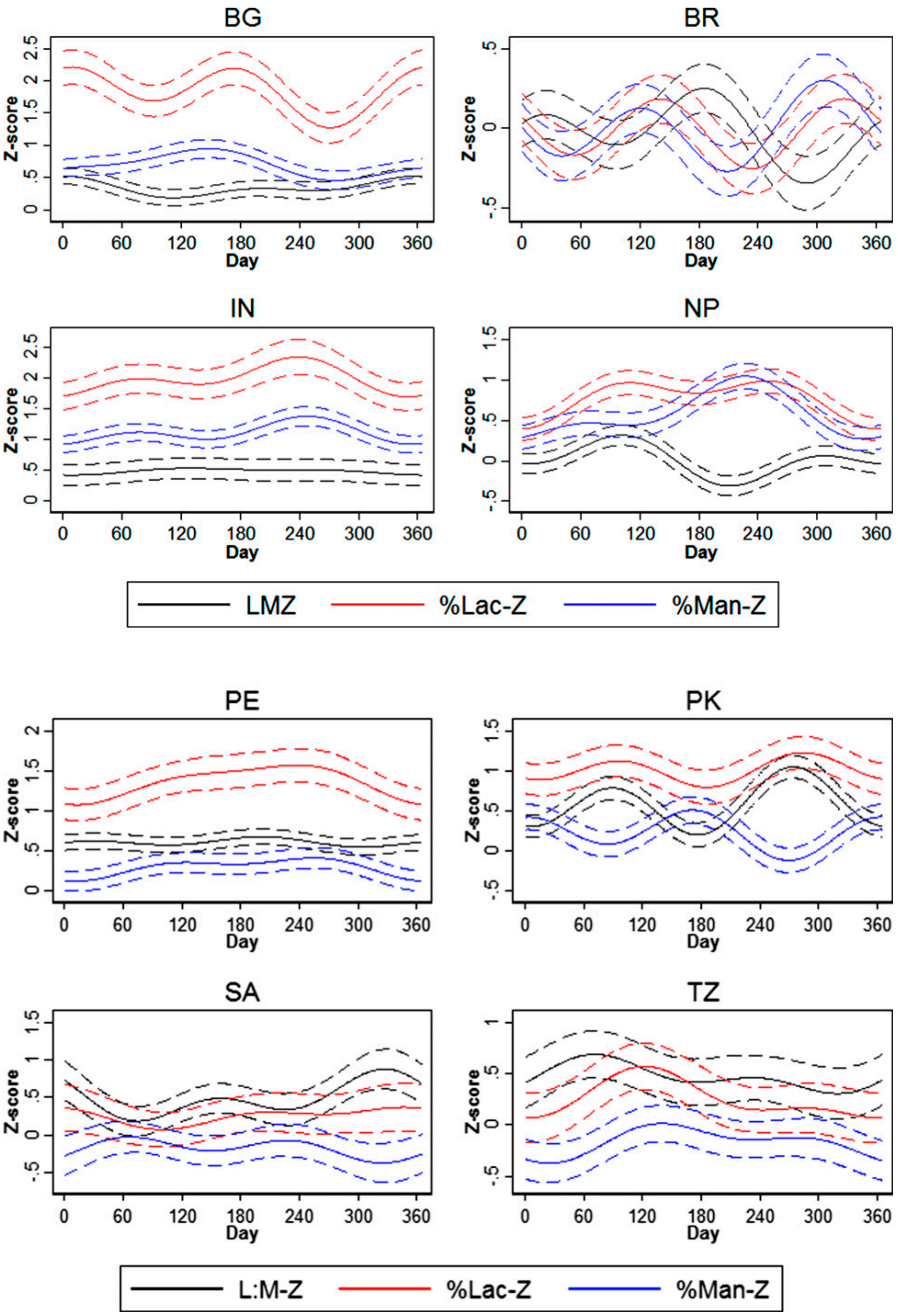

SUPPLEMENTAL FIGURE 1. Modeled seasonal trends in LMZ, %Lac-Z, and %Man-Z for each site. %Lac-Z = percent lactulose excretion, normalized by age and sex and treating the Brazil (BRF) cohort as the reference population; LMZ = L/M ratio, normalized by age and sex and treating the Brazil (BRF) cohort as the reference population; %Man-Z = percent mannitol excretion, normalized by age and sex and treating the Brazil (BRF) cohort as the reference population.

Supplemental Table 1

Overall LMZ models stratified by site, age, and gender

	Overall	BRF laboratory only	BRF	BGD	INV	NEB	PEL	PKN	SAV	TZH	Age = 3 months	Age = 6 months	Age = 9 months	Age = 15 months	Sex = male	Sex = female
WAZ	−0.0518***	−0.0343	−0.00921	−0.145***	−0.0559	−0.0574	−0.0415	−0.0611	0.0435	−0.0431	−0.0665**	−0.0451*	0.00738	−0.0856**	−0.0747***	−0.0234
	(0.000)	(0.063)	(0.808)	(0.000)	(0.187)	(0.086)	(0.051)	(0.068)	(0.399)	(0.430)	(0.009)	(0.038)	(0.773)	(0.002)	(0.000)	(0.189)
Any breastmilk	0.119*	0.276***	0.155	0.903***	−0.372**	0 (.)	0.158	0.541***	−0.111	0.752**	0.445*	−0.121	0.0116	0.306***	0.182*	0.0544
	(0.025)	(0.000)	(0.192)	(0.000)	(0.004)		(0.103)	(0.000)	(0.530)	(0.005)	(0.012)	(0.285)	(0.918)	(0.000)	(0.021)	(0.444)
Any solids	−0.0888*	−0.240***	−0.245	−0.205*	0.181	−0.248*	−0.0645	−0.128	−0.00760	−0.0566	−0.172*	−0.102	−0.216	−0.595	−0.119	−0.0702
	(0.045)	(0.000)	(0.082)	(0.047)	(0.243)	(0.012)	(0.292)	(0.323)	(0.978)	(0.785)	(0.011)	(0.096)	(0.295)	(0.187)	(0.080)	(0.216)
WAMI	−0.392***	−0.348*	−0.296	−0.301	−0.400	−0.555*	−0.333	−0.439*	−0.990*	1.131*	−0.354	−0.0990	−0.342	−0.754***	−0.272	−0.499***
	(0.000)	(0.017)	(0.511)	(0.304)	(0.126)	(0.025)	(0.051)	(0.033)	(0.030)	(0.013)	(0.059)	(0.554)	(0.079)	(0.000)	(0.099)	(0.000)
Pathogens per month	0.0591***	0.0752**	0.0452	0.00383	0.0876	0.0715	−0.0166	0.146***	−0.0338	0.0553	0.0327	0.0495	0.0479	0.0611*	0.0684**	0.0495*
	(0.000)	(0.001)	(0.345)	(0.920)	(0.087)	(0.071)	(0.519)	(0.000)	(0.686)	(0.407)	(0.485)	(0.094)	(0.124)	(0.043)	(0.007)	(0.022)
Pathogens per month (lifetime)	0.0325	0.0486	0.0594	−0.118	0.126	0.0647	0.116	0.0531	0.323 (0.150)	−0.0450	0.0887	−0.0700	0.101	0.0870	−0.0237	0.0848
	(0.425)	(0.376)	(0.618)	(0.241)	(0.331)	(0.564)	(0.093)	(0.515)		(0.769)	(0.271)	(0.310)	(0.225)	(0.322)	(0.707)	(0.103)
Urine volume	0.0538**	0.116***	0.138*	0.0862	−0.165**	0.311***	0.0254	0.0678	−0.148	0.153*	0.0212	0.0731*	0.0839*	0.107**	0.0475	0.0598*
	(0.007)	(0.000)	(0.029)	(0.085)	(0.004)	(0.000)	(0.391)	(0.184)	(0.083)	(0.044)	(0.599)	(0.035)	(0.031)	(0.010)	(0.129)	(0.016)
Site																
BRF	Ref	Ref									Ref	Ref	Ref	Ref	Ref	Ref
BGD	0.109			0 (.)							0.0181	0.299*	0.541***	−0.589***	0.227*	−0.00214
	(0.147)										(0.899)	(0.014)	(0.000)	(0.000)	(0.049)	(0.982)
INV	0.209**				0 (.)						−0.595***	0.217	0.809***	0.342*	0.271*	0.135 (0.168)
	(0.008)										(0.000)	(0.089)	(0.000)	(0.024)	(0.028)	
NEB	−0.0877	−0.106				0 (.)					−0.196	0.00115	0.269*	−0.464***	−0.120	−0.0473
	(0.175)	(0.140)									(0.110)	(0.991)	(0.026)	(0.000)	(0.219)	(0.570)
PEL	0.489***						0 (.)				−0.147	0.485***	1.009***	0.594***	0.620***	0.345***
	(0.000)										(0.264)	(0.000)	(0.000)	(0.000)	(0.000)	(0.000)
PKN	0.334***	0.389***						0 (.)			0.0177	0.523***	0.759***	0.0801	0.431***	0.243*
	(0.000)	(0.000)									(0.906)	(0.000)	(0.000)	(0.585)	(0.000)	(0.013)
SAV	0.445***	0.470***							0 (.)		0.0112	0.481***	0.837***	0.485***	0.480***	0.393***
	(0.000)	(0.000)									(0.933)	(0.000)	(0.000)	(0.001)	(0.000)	(0.000)
TZH	0.146	0.187								0 (.)	0.0494	0.333*	0.650***	−0.425*	0.279*	0.00519
	(0.103)	(0.095)									(0.761)	(0.024)	(0.000)	(0.015)	(0.049)	(0.963)
Month																
3	Ref	Ref	Ref	Ref	Ref	Ref	Ref	Ref	Ref	Ref	0				Ref	Ref
6	0.373***	0.319***	0.110	0.338***	0.488**	0.388***	0.557***	0.365**	0.516**	0.0887		0 (.)			0.330***	0.422***
	(0.000)	(0.000)	(0.420)	(0.000)	(0.002)	(0.000)	(0.000)	(0.006)	(0.001)	(0.628)					(0.000)	(0.000)
9	0.568***	0.454***	0.0583	0.495***	0.854***	0.484***	0.900***	0.453**	0.694***	0.456*			0 (.)		0.566***	0.587***
	(0.000)	(0.000)	(0.696)	(0.000)	(0.000)	(0.000)	(0.000)	(0.001)	(0.000)	(0.018)					(0.000)	(0.000)
15	0.365***	0.228***	0.135	−0.0742	0.755***	0.0678	0.918***	0.347*	0.585***	−0.00894				0 (.)	0.322***	0.413***
	(0.000)	(0.001)	(0.380)	(0.569)	(0.000)	(0.517)	(0.000)	(0.018)	(0.000)	(0.965)					(0.000)	(0.000)
Sex																
Male	Ref	Ref	Ref	Ref	Ref	Ref	Ref	Ref	Ref	Ref	Ref	Ref	Ref	Ref		
Female	−0.0846**	−0.0215	0.0579	−0.184*	−0.133	0.103	−0.177***	−0.116	−0.121	−0.142	−0.209***	−0.0324	−0.112*	−0.0182		
	(0.003)	(0.601)	(0.543)	(0.010)	(0.098)	(0.125)	(0.000)	(0.129)	(0.310)	(0.215)	(0.000)	(0.484)	(0.041)	(0.742)		
Constant	−0.266*	−0.487**	−0.352	−0.793*	0.816*	−0.955***	−0.00423	−0.380	1.378*	−1.017*	−0.0741	0.0516	0.0162	0.556	−0.357	−0.247
	(0.047)	(0.008)	(0.465)	(0.032)	(0.020)	(0.000)	(0.983)	(0.166)	(0.015)	(0.016)	(0.790)	(0.824)	(0.960)	(0.286)	(0.091)	(0.131)
Random effect	0.261***	0.281***	0.224*	0.329***	0.192	0.307***	0.199***	0.190	0.338**	2.99e-15					0.309***	0.196***
	(0.000)	(0.000)	(0.029)	(0.000)	(0.055)	(0.000)	(0.000)	(0.053)	(0.005)	(1.000)					(0.000)	(0.000)
Residual	0.910***	0.965***	0.919***	0.740***	0.987***	0.730***	0.515***	0.868***	1.009***	1.294***					0.986***	0.828***
	(0.000)	(0.000)	(0.000)	(0.000)	(0.000)	(0.000)	(0.000)	(0.000)	(0.000)	(0.000)					(0.000)	(0.000)
Error											0.919***	0.826***	0.955***	0.974***		
											(0.000)	(0.000)	(0.000)	(0.000)		
N	5,362	2,933	486	768	775	813	886	647	417	570	1,315	1,353	1,358	1,336	2,735	2,627
Degrees of freedom	21	18	14	14	14	13	14	14	14	14	18	18	18	18	20	20
AIC	14,047.8	8,027.0	1,343.6	1,835.0	2,085.4	1,949.7	1,529.0	1,623.0	1,151.0	1,785.4	3,511.7	3,307.2	3,491.3	3,521.0	7,469.1	6,513.2

AIC = Akaike information criterion; BGD = Dhaka, Bangladesh; BRF = Fortaleza, Brazil; INV = Vellore, India; LMZ = L/M ratio, normalized by age and sex and treating the Brazil (BRF) cohort as the reference population; NEB = Bhaktapur, Nepal; PEL = Loreto, Peru; PKN = Naushero Feroze, Pakistan; SAV = Venda, South Africa; TZH = TZH; WAMI = Water Assets Maternal education and Income; WAZ = *P* values are given in parentheses.
* $P < 0.05$; ** $P < 0.01$; *** $P < 0.001$.

Supplemental Table 2

Models adjusting for child age, site, gender, and urine volume, as well as the presence of specific enteropathogens at the time of the LM test

	N	%Lac-Z	%Man-Z	LMZ
Asymptomatic adenovirus	145	0.108 (−0.097, 0.314) *P* = 0.302	−0.059 (−0.221, 0.103) *P* = 0.475	0.127 (−0.033, 0.288) *P* = 0.121
Symptomatic adenovirus	26	0.464 (−0.226, 1.154) *P* = 0.187	0.451 (−0.096, 0.997) *P* = 0.106	0.053 (−0.463, 0.569) *P* = 0.839
Asymptomatic astrovirus	133	0.037 (−0.179, 0.253) *P* = 0.738	−0.005 (−0.176, 0.166) *P* = 0.958	0.096 (−0.071, 0.264) *P* = 0.260
Symptomatic astrovirus	50	−0.311 (−0.775, 0.153) *P* = 0.189	−0.271 (−0.631, 0.089) *P* = 0.140	0.258 (−0.101, −0.618) *P* = 0.159
Asymptomatic atypical EPEC	364	−0.055 (−0.187, 0.077) *P* = 0.417	−0.100 (−0.206, 0.006) *P* = 0.065	0.094 (−0.011, 0.198) *P* = 0.078
Symptomatic atypical EPEC	34	−0.130 (−0.643, 0.382) *P* = 0.619	−0.201 (−0.616, 0.214) *P* = 0.343	0.376 (−0.034, 0.787) *P* = 0.073
Asymptomatic *Campylobacter*	1,653	0.048 (−0.029, 0.125) *P* = 0.220	−0.029 (−0.089, 0.032) *P* = 0.353	0.069 (0.010, 0.129) *P* = 0.022
Symptomatic *Campylobacter*	292	−0.010 (−0.207, 0.188) *P* = 0.922	−0.080 (−0.232, 0.073) *P* = 0.307	0.100 (−0.051, 0.0251) *P* = 0.195
Asymptomatic *Cryptosporidium*	229	−0.013 (−0.178, 0.152) *P* = 0.878	−0.181 (−0.314, −0.049) *P* = 0.007	0.242 (0.111, 0.373) *P* < 0.001
Symptomatic *Cryptosporidium*	55	0.407 (−0.090, 0.905) *P* = 0.109	0.117 (−0.250, 0.483) *P* = 0.532	0.095 (−0.270, 0.461) *P* = 0.610
Asymptomatic EAEC	1,800	−0.023 (−0.092, 0.047) *P* = 0.520	0.002 (−0.054, 0.058) *P* = 0.951	−0.018 (−0.072, 0.037) *P* = 0.522
Symptomatic EAEC	209	−0.030 (−0.261, 0.201) *P* = 0.708	−0.072 (−0.256, 0.112) *P* = 0.441	0.072 (−0.109, 0.253) *P* = 0.437
Asymptomatic EPEC	230	−0.022 (−0.189, 0.145) *P* = 0.795	−0.033 (−0.166, 0.100) *P* = 0.630	0.001 (−0.128, 0.130) *P* = 0.989
Symptomatic EPEC	45	0.006 (−0.481, 0.493) *P* = 0.981	0.221 (−0.161, 0.603) *P* = 0.257	−0.291 (−0.659, 0.077) *P* = 0.121
Asymptomatic *Giardia*	657	0.169 (0.057, 0.282) *P* = 0.003	−0.062 (−0.150, 0.026) *P* = 0.169	0.226 (0.139, 0.313) *P* < 0.001
Symptomatic *Giardia*	96	0.175 (−0.182, 0.532) *P* = 0.336	0.002 (−0.272, 0.277) *P* = 0.986	0.228 (−0.042, 0.499) *P* = 0.098
Asymptomatic LT-ETEC	285	−0.014 (−0.0161, 0.134) *P* = 0.857	−0.138 (−0.256, −0.020) *P* = 0.022	0.114 (−0.002, 0.231) *P* = 0.055
Symptomatic LT-ETEC	47	0.056 (−0.419, 0.531) *P* = 0.818	−0.188 (−0.558, 0.182) *P* = 0.319	0.266 (−0.111, 0.642) *P* = 0.166
Asymptomatic ST-ETEC	114	0.131 (−0.101, 0.363) *P* = 0.268	0.050 (−0.135, 0.234) *P* = 0.598	−0.000 (−0.180, 0.179) *P* = 0.998
Symptomatic ST-ETEC	32	0.269 (−0.306, 0.842) *P* = 0.359	−0.174 (−0.597, 0.249) *P* = 0.421	0.474 (0.045, 0.904) *P* = 0.030

LM test = the lactulose mannitol test; %Lac-Z = Percent lactulose excretion, normalized by age and sex and treating the Brazil (BRF) cohort as the reference population; LMZ = L/M ratio, normalized by age and sex and treating the Brazil (BRF) cohort as the reference population; %Man-Z = Percent mannitol excretion, normalized by age and sex and treating the Brazil (BRF) cohort as the reference population. "Asymptomatic" here refers to the pathogen detected in the concurrent asymptomatic stool. "Symptomatic" refers to the presence of any diarrhea associated with the pathogen within the 0–14 days prior to the LM test. The top 10 pathogens (by asymptomatic prevalence prior to the LM test) are shown here.
